# Physical Activity Patterns After Retirement: The REGARDS Study

**DOI:** 10.1093/geroni/igaa057.1704

**Published:** 2020-12-16

**Authors:** Eric Shiroma, J David Rhodes, Aleena Bennet, Monika M Safford, Leslie MacDonald, Steven P Hooker, Virginia Howard

**Affiliations:** 1 National Institute on Aging, Bethesda, Maryland, United States; 2 University of Alabama at Birmingham, Birmingham, Alabama, United States; 3 Weill Cornell Medical College, New York, New York, United States; 4 National Institute for Occupational Safety and Health, Cincinnati, Ohio, United States; 5 San Diego State University, San Diego, California, United States

## Abstract

Major life events, such as retirement, may lead to dramatic shifts in physical activity (PA) patterns. However, there are limited empirical data quantifying the magnitude of these changes. Our aims were to objectively measure PA before and after retirement and to describe changes in participation in various types of PA. Participants were employed black and white men and women enrolled in REGARDS (REasons for Geographic and Racial Differences in Stroke), a national prospective cohort study (n=581, mean age 64 years, 25% black, 51% women). Participants met inclusion criteria if they retired between their first and second accelerometer wearing (2009-2013 and 2017-2018, respectively) and had valid accelerometer data (>4 days with >10 hours/day pre- and post-retirement). Accelerometer-based PA was categorized into average minutes per day spent in sedentary, light-intensity, and moderate-to-vigorous PA. Participants reported changes (less, same, more) in 12 types of PA. After retirement, participants decreased both sedentary time (by 36.3 minutes/day) and moderate-to-vigorous PA (by 5.6 minutes/day). Conversely, there was an increase in light-intensity PA (+18.1 minutes/day) after retirement. Participants reported changes in their participation level in various PA activities. For example, 41% reported an increased amount of TV viewing, 42% reported less walking, and 31% reported increased participation in volunteer activities. Findings indicate that retirement coincides with a change in the time spent in each intensity category and the time spent across a range of activity types. Further research is warranted to examine how these changes in physical activity patterns influence post-retirement health status.

